# Subgingival areas as potential reservoirs of different *Candida* spp in type 2 diabetes patients and healthy subjects

**DOI:** 10.1371/journal.pone.0210527

**Published:** 2019-01-10

**Authors:** Sanja Matic Petrovic, Milena Radunovic, Milena Barac, Jovana Kuzmanovic Pficer, Dusan Pavlica, Valentina Arsic Arsenijevic, Ana Pucar

**Affiliations:** 1 Department of Oral Medicine and Periodontology, School of Dental Medicine, University of Belgrade, Belgrade, Serbia; 2 Department of Microbiology, School of Dental Medicine, University of Belgrade, Belgrade, Serbia; 3 Department for Medical Statistics and Informatics, School of Dental Medicine, University of Belgrade, Belgrade, Serbia; 4 Department of Microbiology and Immunology, School of Medicine, University of Belgrade, Belgrade, Serbia; University of the Pacific, UNITED STATES

## Abstract

**Objectives:**

The aim of this cross-sectional observational study was to compare the prevalence of different oral *Candida* spp. in patients with Type 2 Diabetes and chronic periodontitis in two oral sites: dorsal surface of the tongue and subgingival area. In order to determine subgingival areas as potential reservoirs of yeasts, this study aimed to find differences in the yeasts’ detection between the dorsum of the tongue, as the oral site most commonly inhabited with microorganisms, and subgingival samples. Additionally, potential predictors for the yeasts prevalence were determined.

**Material and methods:**

Subjects (N = 146) were divided into four groups: group A- healthy individuals without periodontitis, group B- healthy individuals with chronic periodontitis, group C- Type 2 Diabetes patients with good glycoregulation and Chronic periodontitis and group D- Type 2 Diabetes patients with poor glycoregulation and Chronic periodontitis. Samples were obtained from the tongue by swabbing. Subgingival plaque samples were taken by paper points and periodontal curette. Isolation and identification of different *Candida* spp. was done using ChromAgar medium. In addition, germ-tube production and carbohydrate assimilation tests were performed.

**Results:**

The prevalence of *Candida* spp. was higher in diabetics with poor glycoregulation. The most frequently isolated species was *Candida albicans* followed by *Candida glabrata* and *Candida tropicalis*. In 15.6% of cases, *Candida* spp. was present in the subgingival area while absent on the tongue. Multivariate regression model showed that HbA1c was *Candida* spp. predictor for both locations.

**Conclusions:**

Our results confirmed that there are *Candida* spp. carriers among subjects with clinically healthy oral mucosa. Also, this study identified subgingival areas as potential reservoirs of these pathogenic species. Glycoregulation has been recognized as a positive predictor factor of *Candida* spp.

## Introduction

Chronic periodontitis (CP) and oral mucosal candidiasis have been considered as chronic complications of Type 2 Diabetes (T2D) [1, 2]. Immunocompromised patients (e.g. Diabetics, AIDS patients) are more prone to *Candida* colonization and infection [1, 3–5]. In the oral cavity, *Candida* spp. is most commonly found on the dorsal surface of the tongue, followed by the palatal and buccal mucosa [6]. However, yeasts have also been found in subgingival areas [7]. Bacteria in the dental biofilm are thought to be the main etiological factor of periodontitis, but there is an growing evidence about the importance of viruses [8] and yeasts [9] in pathogenesis of chronic periodontitis. There is still a disagreement if the yeasts are transient members of oral biofilms [10], or if they are definite members of the oral microbiome [11]. Subgingival yeasts are present in 10–30% of healthy subjects [11, 12] and in up to 52% of diabetics [11, 13, 14].

Although *Candida albicans* is the most frequent species of *Candida* genus, the prevalence and importance of other species, such as *Candida glabrata*, *Candida tropicalis*, *Candida krusei* and *Candida dubliniensis* are increasing [4]. These, non-albicans species were first considered as markers of immunocompromised subjects, but have recently also been isolated from healthy subjects [3, 11].

Since antibiotics are commonly used in the treatment of periodontitis, allowing yeasts to proliferate freely, the possible role of yeasts in the pathogenesis of periodontal diseases is important, especially for immunocompromised subjects.

The purpose of this study was to compare the frequency of different *Candida* spp. on the tongue and in the subgingival sites between healthy subjects and T2D patients with chronic periodontitis. In order to determine subgingival areas as potential reservoirs of yeasts, this study aimed to find differences in the yeasts’ detection between the dorsum of the tongue, as the oral site most commonly inhabited with microorganisms, and subgingival samples. Additionally, potential predictors for the yeasts prevalence were determined.

## Material and methods

### Study population and study design

**Fig 1:**
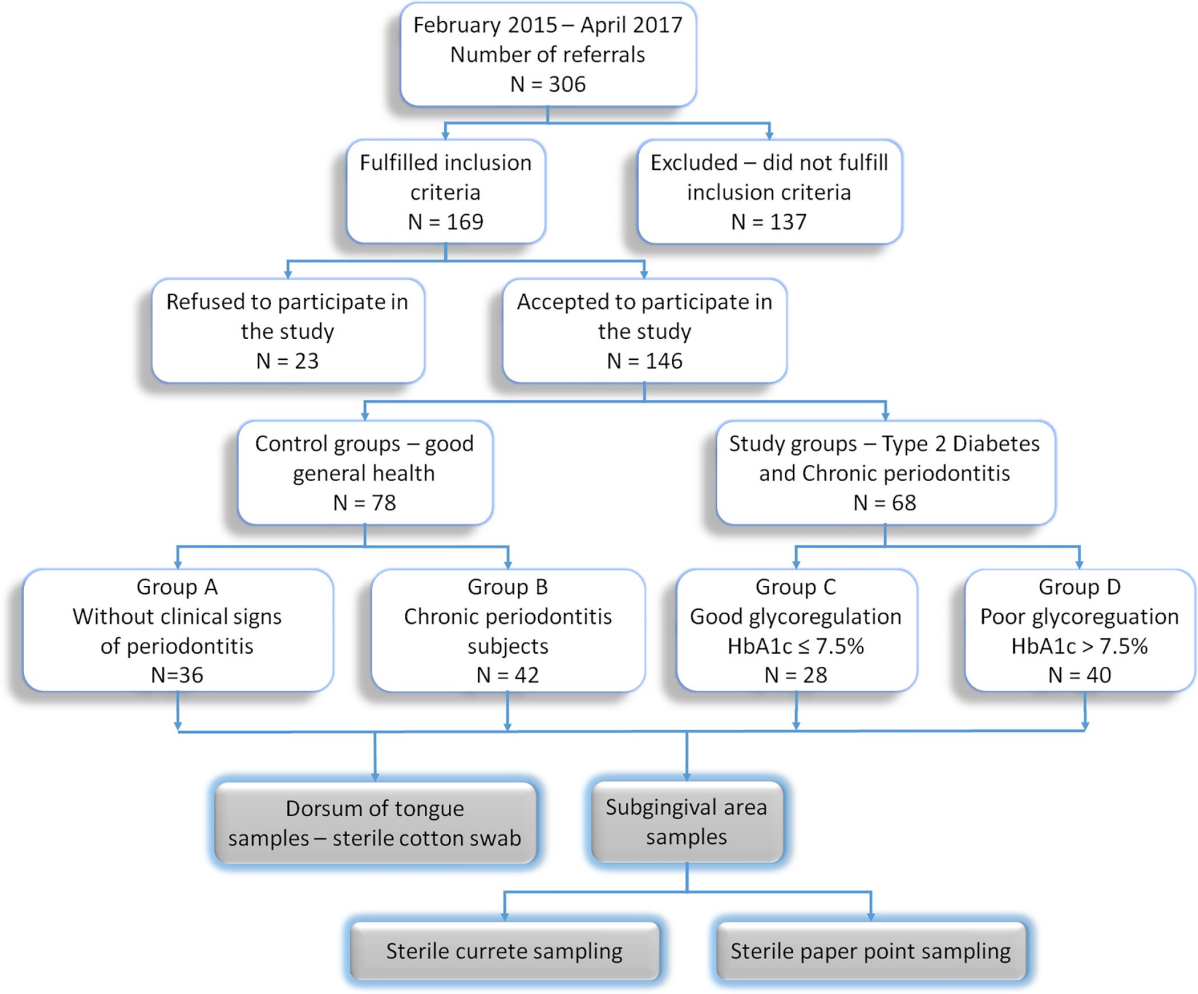
Study and subject design.

This cross-sectional observational study, conducted from February 2015 to April 2017, has the approval of Ethical Committee of the School of Dental Medicine, University of Belgrade in accordance with the Declaration of Helsinki (Ethics Approval no. 36/8, 20 Feb 2015). A total of 146 subjects, who signed an informed consent, were selected and classified in four -experimental groups (Fig 1):

- Group A: 36 healthy volunteers without clinical signs of periodontitis (Probing pocket depth, PPD < 3 mm, Clinical Attachment Loss, CAL = 0 mm, Bleeding on Probing, BOP < 25%)
- Group B: 42 patients with diagnosed CP and free of systemic diseases. Generalized CP was diagnosed if the subject exhibited CAL ≥ 1 mm and PPD > 3 mm at >30% of sites. According to CAL, periodontitis was defined as slight (CAL = 1-2mm), moderate (CAL = 3-4mm) or severe (CAL>5mm) [15]. Patients with periodontitis were referred to the Department of Periodontology and Oral Medicine, School of Dental Medicine, University of Belgrade.
- Group C: 28 patients with diagnosed CP and T2D with satisfactory metabolic control (HbA1c ≤ 7.5%). T2D was diagnosed by measuring glycated hemoglobin (HbA_1_c) and blood glucose levels (using oral glucose tolerance test) [16].
- Group D: 40 patients with diagnosed CP, T2D and with poor metabolic control (HbA1c > 7.5%). Diabetic patients (C and D groups) were referred to the Clinic for Endocrinology, Diabetes and Metabolic Diseases, Clinical Center of Serbia.

### Inclusion and exclusion criteria

In order to take part in the study, the subjects were expected to have more than 14 teeth, and not show any of the following traits: presence of any disease except T2D and its complications, aggressive periodontitis, therapy that could affect the periodontium (antibiotics, topical or systematic corticosteroids or antiseptics), previous oral candidiasis treatment, pregnancy/lactation and periodontal treatment in the last 1.5 year.

### Socio-demographic and lifestyle data, clinical and microbiological outcomes and additional measures

Each patient had undergone 1) socio-demographic and lifestyle data using questionnaire interview; 2) clinical evaluation and measurements (secondary outcomes); 3) microbiological sampling procedures (primary outcomes) and 4) additional measurements.

1) Socio-demographic and lifestyle data using questionnaire interview included age, gender and education data. Also, parameters that could potentially influence clinical parameters of periodontitis and/or T2D or Candida carriage, but which could not be regarded as exclusion criteria were recorded: self-reported information about xerostomia, blood type, everyday intake of sweets and smoking habits (“non-smokers” and “smokers”). Patients who ceased smoking less than 6 months prior to the study were not included

2) Clinical evaluation and measurement (a full mouth clinical examination) was performed by two experienced and calibrated doctors, using Williams’ probe calibrated in millimeters (Hu-Friedy Chicago, IL). The following periodontal parameters at six sites per tooth were assed: 1) plaque index-Silness Loe (PI), index of oral hygiene based on recording both soft debris and mineralized deposits on tooth, 2) bleeding on probing (BOP), measured 15 seconds after probing, defined as presence or absence, 3) probing pocket depth (PPD) as a distance from a margin of gingiva to the bottom of the sulcus/pocket (expressed in mm), 4) clinical attachment level (CAL) as distance from cemento-enamel junction to the pocket bottom (expressed in mm). The PPD and CAL were rounded to the higher millimeter and done at all teeth except third molars. For the evaluation of intra-examiner agreement, 20% of samples were reexamined 2 days after the first examinations.

3) Microbiological sampling procedures started a day after periodontal examination. Oral swabs were collected using a sterile cotton stick by swabbing ten times from the dorsum of the tongue and immediately inoculated on Sabouraud dextrose agar (Oxoid, Basingstoke, UK). The subgingival samples were obtained from the tooth with deepest PPD. First, cotton rolls were used to isolate the tooth and the supragingival plaque was removed by a curette and sterile gauze. Two sterile paper points (#30) were placed into the pocket/sulcus until a mild resistance appeared and kept for 30 seconds. Paper points contaminated by blood were excluded. Immediately after, sterile curette (S4L/4R SS G.Hartzell&Son, Concord, California) was used to obtain samples of the complete subgingival biofilm. Both subgingival samples were inoculated in separate sterile plastic tubes containing 1 mL of Sabouraud dextrose broth, vortexed for 60 seconds. From each sample, 20μl of suspended broth was streaked on SDA using sterile plastic micro pipette and incubated at 37°C for 48 hours. The number of Colony Forming Units per milliliter (CFU/ml) for samples taken by paper points was counted by one calibrated microbiologist. Detailed protocol can be found at http://dx.doi.org/10.17504/protocols.io.wdefa3e. The same microbiologist defined growth for samples taken from tongue as rare (number of CFU per sample ≤ 5), medium (6–50 CFU per sample) and dense (number of CFU per sample > 50).

Distinction of different species of *Candida* genus was done using CHROMagar Candida Medium (Becton Dickinson) according to producers instructions, germ-tube production test and carbohydrate assimilation test [17]. After identification of more than one Candida species per sample, the levels of each species were defined as percentage of overall culture growth. The microbiologist was blinded for any clinical and anamnestic information.

After cultivation or second clinical examination (for examination of intra rater reliability), subjects received necessary dental treatment.

4) Additional measurements include biochemical and hematological analysis: fasting plasma glucose levels (FPG), HbA1c, hematological parameters: red blood count (RBC), hemoglobin (Hgb), hematocrit (HCT), mean corpuscular volume (MCV), mean cell hemoglobin (MCH), mean corpuscular hemoglobin concentration (MCHC) and sedimentation rate.

### Statistical analysis

SPSS 22.0 software package for Windows (SPSS inc. Chicago, USA) was used for statistical analysis. Descriptive data were presented as Mean (SD) for numerical or the percentage for discrete measures. ANOVA with Bonferroni correction was used for normally distributed data. Non-parametric data was analyzed using the Kruskall-Wallis and Mann-Whitney test. Chi Square Test (χ2) was used for comparison of categorical variables. The logistic regression model was used to determine predictors of the presence of *Candida spp*. Subjects with missing data were not included in the study. Inter-rater reliability was appraised for each periodontal clinical parameter using the Cohen’s kappa statistics. Differences were considered significant when p-value was < 0.05.

## Results

### Socio-demographic, clinical outcome variables and additional measurements

According to results from questionnaires, we found that groups were similar by age, gender and smoking habits (Table 1). Also, subjects with O vs. A/AB/B blood type system were equally distributed between groups (Table 1).

**Table 1:** Demographic records, blood count and biochemical analysis data in groups.

		Groups				p value
		A (N = 36)	B (N = 42)	C (N = 28)	D (N = 40)	
Socio-demographic data						
Gender N(%) m / f		15(41.7) / 21(58.3)	17(40.5) / 25(59.5)	15(53.6) / 13(46.4)	26(65.0) / 14(35.0)	†.099
Age (x±SD)		43±4	48±11	48±7	47±7	‡.053
Smokers N (%)		8(22.2)	16(38.1)	9(32.1)	10(25.0)	†.408
Blood type sistem N (%)	O	5 (13.9)	8 (19.0)	6 (21.4)	4 (10.0)	†.549
	A/B/AB	31 (86.1)	34 (81.0)	22 (78.6)	36 (90.0)	
Clinical periodontal parameters (presented as Mean±SD)						
Teeth number		27.58±1.422	20.36±5.28	16.89±4.646	14.85±3.899	§ < .0001*
PI		0.86±0.468	2.38±3.737	2.68±0.450	2.24±0.751	§ < .0001*
BOP (%)		39.58±19.099	61.24±24.390	62.58±25.370	64.40±28.888	‡ < .0001*	
PPD (mm)		2.02±0.513	2.93±0.871	2.87±0.903	2.71±0.757	‡ < .0001*
CAL (mm)		NA	3.37±1.964	3.87±1.929	4.13±2.103	‡.233
Additional (biochemical and hematological) measurements (presented as Mean±SD)						
FPG (mmol/L)		4.64±0.534	4.97±0.578	7.59±1.824	11.24±4.061	§ < .0001*
HbA_1c_ (%)		4.80±0.607	4.82±0.561	7.10±0.566	10.81±1.357	§ < .0001*
Hgb (g/l)		137.08±10.308	114.81±52.717	139.75±11.670	137.19±14.731	§.355
RBC (10^12^/l)		4.61±0.474	4.31±1.197	4.62±0.375	4.69±0.601	§.458
MVC (fl)		89.15±9.299	91.82±9.857	89.15±3.620	88.78±9.686	§.567
HCT (l/l)		0.409±0.054	0.690±0.595	0.420±0.034	0.415±0.064	§.045*
MCH (pg)		29.91±2.565	28.82±2.747	30.27±1.489	29.43±2.511	‡.074
MCHC (g/l)		337.14±31.198	316.45±40.295	339.39±15.908	334.28±39.350	§.021*

X = mean; SD = standard deviation; m/f = males/females; PI = Silness Loe Plaque Index; BOP = bleeding on probing index; PPD = Probing Pocket Depth; CAL = Clinical Attachment Loss; FPG = Fasting plasma glucose level; HbA_1c_ = glycolisated hemoglobin; Hgb = Hemoglobin; RBC = Red Blood Count; MCV = Mean Corpuscular Volumen; HCT = Hematocrit; MCH = Mean Cell Hemoglobin; MCHC = mean corpuscular hemoglobin concentration

† Chi Square Test

‡ANOVA

§Kruskall-Wallis Test

*results with statistical significance

Clinical examination showed that groups differed according to the number of present teeth (Table 1). Intergroup analysis showed that similar number of teeth was found only between groups C and D (Man-Whitney U Test, p = 0.073), while other groups exhibited different number of teeth (Man-Whitney U Test, A vs. B: p<0.0001, A vs. C: p<0.0001, A vs. D: p<0.0001, B vs. C: p = 0.009, B vs. D: p<0.0001). PI was the highest in diabetics with good glycoregulation, and differed between all groups (Mann-Whitey U test, p < 0.05 for all intergroup comparisons). BOP was significantly higher in groups B, C and D than in group A (ANOVA with Bonferroni correction, p = 0.001, p = 0.002 and p<0.0001, respectively). PPD differed between A vs. B, A vs. C and A vs. D groups (Bonferroni p<0.0001 for all three intergroup comparisons) Mean PPD values were similar between other groups (B vs. C: p = 1.000, B vs. D: p = 1.000, C vs. D: p = 1.000). CAL was similar among three groups of patients with diagnosed periodontitis (Table 1). Kappa scores were 0.5–0.6 for inter-rater agreement.

As expected, FPG level was statistically different between all groups, the highest being in the group of subjects with poorly regulated blood glucose level (Mann-Whitney U Test, p<0.05 for all comparisons) (Table 1). HbA1C level was highest in group D, followed by group C. It differed between all groups except A vs. B (Mann-Whitney U Test, p = 0.903) (Table 1). Results of hematological measurements for Hgb, RBC, MCV and MCH were similar among groups (Kruskall-Wallis Test, p>0.05 for all parameters). HCT was statistically different only between groups A and B (Mann-Whitney U Test, p = 0.011). MCHC values differed between groups B and C (Mann-Whitney U Test, p = 0.011) and groups C and D (Mann-Whitney U Test, p = 0.016).

### Microbiological (primary) outcome variables

**Fig 2:**
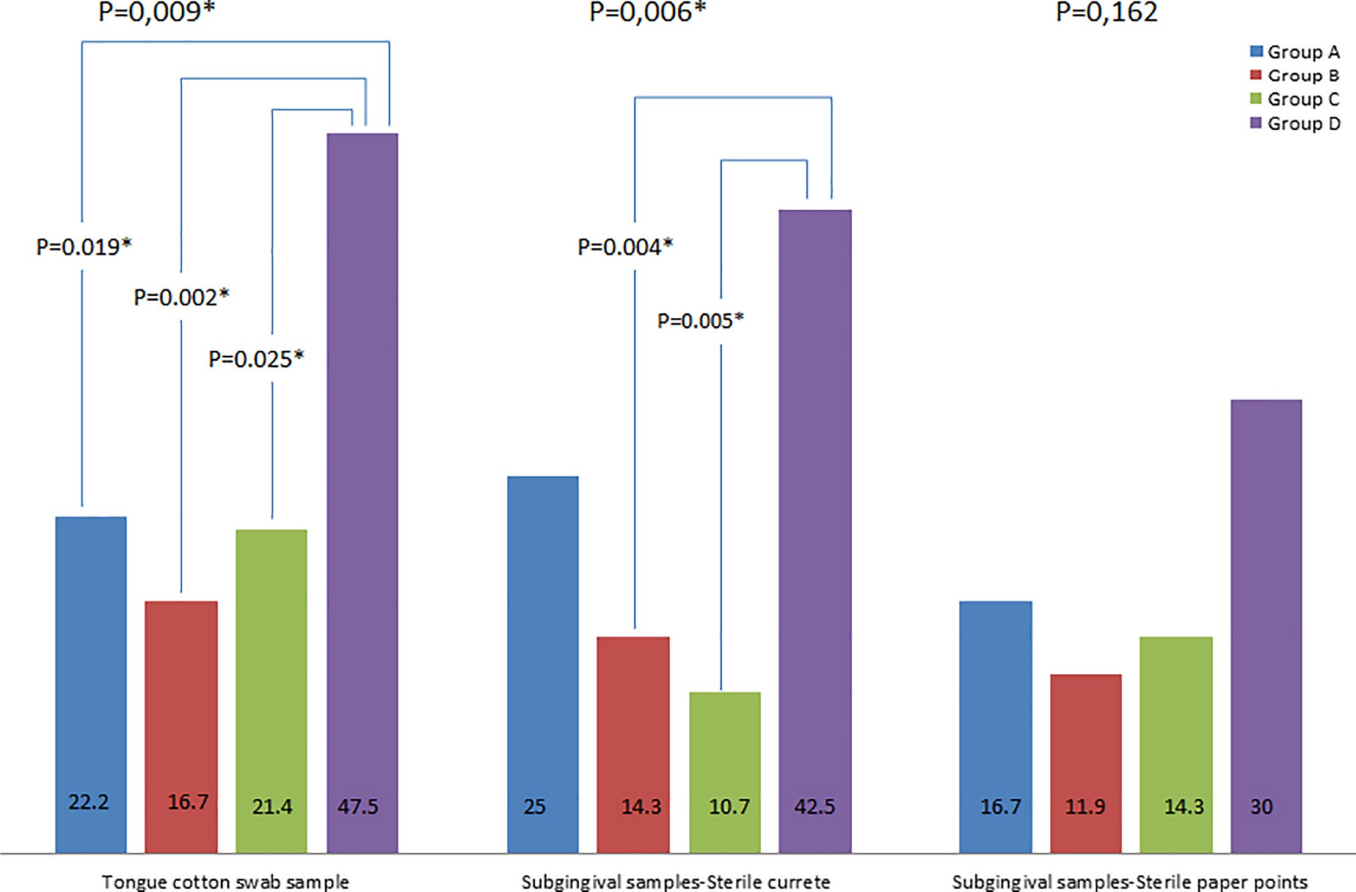
Positive findings of Candida spp. on tongue and in subgingival areas (presented as %). * presents statistical significant difference (p<0.05).

*Candida* spp. was detected on the tongue of 27.4% of subjects. It was more prevalent in diabetics with poor glycoregulation (47.5%) than in groups A (22.2%), B (16.7%) or C (21.5%) (Fig 2). Subjects with T2D (C+D = 25) had a significantly higher prevalence of *Candida* spp. detection (37.3%), than healthy subjects (A+B = 15 (19.5%)) (p = 0.013). The frequency of isolation of more than one *Candida* species (mixed isolation) per sample was higher in group D than in group A (p = 0.002) and group B (p = 0.018) (Table 2). The most frequently isolated species in this study was *C*. *albicans—*detected in all positive samples (27.4%), followed by *C*. *glabrata* (6.85%) and *C*. *tropicalis* (1.37%). In the samples of mixed infection of tongue, *Candida albicans* was the predominant species, presenting 75.83±19.287% (mean±SD) of isolates per sample. Even though there was no statistically significant difference in the amount of detected yeasts on tongue among groups (p = 0.331), it is interesting that group A presented only rare growth, while dense growth was detected only in group D.

**Table 2:** Candida spp. finding on tongue and in subgingival areas sampled by sterile periodontal curette and sterile paper point.

**Tongue samples (sterile cotton swab)**					
	Group A (N = 36)	Group B (N = 42)	Group C (N = 28)	Group D (N = 40)	P value
Negative yeast finding N (%)	28 (77.8%)	35 (83.3%)	22 (78.5)	21 (52.5)	†0.009*
Positive yeast finding N (%)	8 (22.2%)	7 (16.7%)	6 (21.5)	19 (47.5)	
Non albicans finding: N (%)	0 (0)	1 (2.4%)	2 (7.1)	9(22.5)	
**Sterile currette sample**					
	Group A	Group B	Group C	Group D	P value
Negative yeast finding N (%)	27 (75%)	36 (87%)	25 (89%)	23 (57%)	†0.007*
Positive yeast finding N (%)	9 (25%)	6 (13%)	3 (11%)	17 (43)	
Non albicans finding: N (%)	1 (2.8)	1 (2.4)	1 (3.6)	5(12.5)	
**Sterile paper points samples**					
	Group A	Group B	Group C	Group D	P value
Negative yeast finding N (%)	30 (83%)	37 (88%)	24 (85%)	28 (70%)	†0.162
Positive yeast finding N (%)	6 (17%)	5 (12)	12 (30%)	12 (30%)	
Non albicans finding: N (%)	0 (0)	1 (2.4)	2 (7.1)	4 (10.0)	

† Chi Square Test

*results with statistical significance

In the samples obtained by sterile periodontal curette, 23.97% were positive (Fig 2). The frequency of *Candida* spp. isolation was higher in group D than in group B and group C. The most frequently isolated species was *C*. *albicans* (23.97%), followed by *C*. *glabrata* (5.48%). The frequency of mixed isolation of *Candida* spp. was similar between the groups (p = 0.152) (Table 2). In the samples of mixed infection, *Candida albicans* was the predominant species, presenting 76.25±19.226% (mean±SD) of isolates per sample.

*Candida* spp. was isolated in 18.49% of subgingival samples collected by sterile paper points (Fig 2). The most frequently isolated species was *C*. *albicans* (18.49%), followed by *C*. *glabrata* (4.79%) and *C*. *tropicalis* (0.80%). In the mixed infection of paper point’s samples, *Candida albicans* was the predominant species, presenting 75.72±20.702% (mean±SD) of isolates per sample. The frequency of mixed isolation of *Candida* spp. was similar between the groups (p = 0.167) (Table 2).

The presence of *C*. *albicans* in subgingival areas detected by either sampling method was 29.25%. Occurrence of *Candida* spp. in group D was significantly higher than in group A (p = 0.018), group B (p = 0.023) and group C (p = 0.005). Within each group, there was no difference in clinical periodontal parameters (PI, BOP, PPD, CAL) between patients with and without *Candida* spp. (p > 0.05 for all comparisons). Frequency of subgingival *Candida* detection differed according to sampling technique (p<0.0001).

The number of subgingival CFU/ml did not differ between groups (Kruskal-Wallis, p = 0.290). The highest values for CFU/ml were detected in group B (1290.00±1281.77), followed by group C (875.48± 485.63), group D (772.98± 659.43) and group A (478.57± 654.38) (expressed as mean±SD).

There were 15.07% of cases where yeasts were not detected on the tongue, but were found in the subgingival area. The frequencies of such findings were similar among groups (p = 0.846): A = 4/36 (11.1%), B = 6/42 (14.3%), C = 5/28 (17.9%), D = 7/40 (17.5%). Only 2.1% of subjects had different species of *Candida* present on tongue and in subgingival area, distributed similarly in groups B, C and D. On the other hand, only in 13.7% of subjects, both samples were positive.

### Logistic regression analysis of the relationship among Candida carriage risk factor and prevalence of *Candida* spp. on tongue and in subgingival sites

Using logistic regression model, age, gender, blood type, smoking habits, presence of PD, presence of T2D, mentioned hematological and biochemical parameters were analyzed as potential predictors of Candida presence. FPG (OR = 1.138, CI = 1.031–1.256, p = 0.011) and HbA1c (OR = 1.273, CI = 1.112–1.459, p<0.0001) have been shown as significant confounders. Only HbA1c (OR = 1.242, CI = 1.011–1.525, p = 0.039) was an independent predictor of the presence of *Candida* spp. using the multivariate logistic regression model.

The univariate logistic regression model was also applied in order to identify parameters that could predict positive detection of *Candida spp*. in the subgingival areas. Age, gender, blood type, smoking habits, presence of periodontitis or T2D, measured hematological and biochemical parameters and periodontal clinical parameters (PI, PPD and CAL) were analyzed as potential predictors. Although FPG (OR = 1.103, CI = 1.002–1.215, p = 0.045), Hb1c (OR = 1.242, CI = 1.087–1.415, p = 0.001) and RDW (OR = 1.219, CI = 1.009–1.474, p = 0.040) were confounders in univariate logistic regression analysis, only HbA1c was the confounder in multivariate regression model (OR = 1.290, CI = 1.062–1.567, p = 0.010).

## Discussion

Although isolation of *Candida* spp. from the oral cavity does not mean disease per se, opinions about the role of yeasts isolated from periodontal pockets vary [10, 11]. Diabetic patients are more susceptible to fungal carriage and infections [1, 18], as well as periodontitis commonly treated by antibiotics as adjuvant periodontal therapy. Therefore, the presence and role of yeasts in pathogenesis of periodontitis in immunocompromised subjects should be clarified.

The prevalence of oral yeasts in this study was determined using sterile cotton swabs of the middle and posterior thirds of the tongue midline, as it has been proven that *Candida* spp. most commonly inhabits these regions in the oral cavity [6]. This prevalence in our study was the highest in diabetics with poor glycoregulation, followed by diabetics with satisfactory glycoregulation, which is in concurrence with previously conducted studies [3, 19]. It is interesting that we found that the prevalence of *Candida* spp. on the tongue of diabetics with satisfactory glycoregulation was similar to the prevalence in both groups of healthy subjects. Bader et al. similarly concluded that good control of blood glucose level is the best protection against *Candida* infection [19]. Additionally, among a number of examined factors in this study, only HbA1c- parameter of glycoregulation has been proven to be an independent risk factor for positive yeast detection on the tongue. The prevalence of *Candida* spp. on the tongue in healthy patients is similar to another study exploring the frequency of this yeast on tongue of healthy subjects in Serbia [20]. Similar to the findings of Zomorodian et al. [3], *C*. *albicans* was the most frequent found species, followed by *C*. *glabrata*, and *C*. *tropicalis*- the last one isolated only at diabetics. Recovery of non- albicans species is important because of their high level of resistance to antifungal drugs [21].

Overall prevalence of subgingival *Candida* spp. was 29.25%. There is no standardized methodology for sampling the subgingival plaque, so in this study collection of subgingival biofilm was done by two commonly used techniques: paper points followed by sterile periodontal curette. Using both methodologies, we demonstrated that there is difference in efficiency of sampling methods. These results should encourage researchers to elucidate optimal methodology for sampling subgingival plaque as our research group started [22]. It is important to emphasize that our previously conducted study has been done at another study population. Results of this study showed higher prevalence of *Candida* spp. in subgingival areas of poorly regulated diabetic subjects than in groups A, B and C. Additionally, our results showed that HbA1c is the only independent predictor for detection of subgingival *Candida* spp. These results are in agreement with already mentioned data about prevention of *Candida* infection by good glycoregulation [13, 19]. Other studies showed occurrence of subgingival *Candida* spp. [11] in 10% of subjects with periodontal health, 30–44% in periodontitis patients [11, 14], and 35.2% to 52% in diabetics [13, 14]. In this study, subjects with periodontal health showed higher prevalence of subgingival yeasts (22.2%), but on the other hand periodontitis patients showed lower prevalence than other studies (14.6%). These surprisingly uncommon results may be explained by proofs about *in vitro* inhibitory effects of cariogenic (e.g. *Streptococcus mutans*) and periopathogenic (eg. *Prevotela intermedia*) bacteria on the growth of *Candida* spp. within biofilms [23], and oral microbiome interactions between Candida and other residents of oral mucosa [5]. Although we did not determine the presence of other microorganisms in subgingival areas, it can be assumed that there are more periopathogenic microorganisms in periodontal pockets than in the gingival sulci. The majority of studies did not divide patients according to glycoregulation when examining the prevalence of yeasts. The group with satisfactory glycoregulation showed similar prevalence to patients with periodontitis only, while subjects with poorly regulated blood glucose level showed higher prevalence. Although there are few of studies analyzing the presence of yeasts in subgingival areas, to the best of our knowledge, our study is of unique design. We considered clinical periodontal status in subjects without metabolic disorders and on the other hand, in subjects with T2D and CP, we considered glycoregulation. Even though we planned a group of patients with T2D and clinically healthy periodontium, we could not find any subjects who fitted these criteria, so we omitted this group.

Comparable to other studies, *C*. *albicans* was the most frequently isolated species [11, 13], but unlike these studies we did not find *C*. *dubliniensis* in subgingival samples. The frequency of *C*. *glabrata* was similar to mentioned studies. *C*. *tropicalis* was isolated only in T2D patients, which is in agreement with the fact that this species is commonly isolated from immunocompromised patients [24] and not from healthy ones [3]. Some researches consider *C*. *tropicalis* as the third most commonly isolated non-albicans species, which is associated with higher mortality than other non-albicans species [24], and has ability to develop rapid resistance to fluconazole [25], so recognition of potential reservoirs of this species on clinically healthy tongue or in periodontal pockets could be useful. Similarly, *C* .*glabrata* is also considered as second or third most common isolate of *Candida* spp. [26], and is increasingly connected with mucosal and general infections, especially in diabetics [27]. In our study, it has also been found in healthy subjects with periodontitis, but even in one sample from clinically healthy periodontium (isolated only by sterile curette). Since periodontitis itself has been defined as a state of disturbed cellular and humoral immune local response [28], it can be expected for *C*. *glabrata* to be found in periodontal pockets. It should be stressed that colonization does not necessarily indicate candidiasis, so *C*. *glabrata* in gingival sulcus can be observed as transient member or possibly a reservoir of yeasts. Also, these two species have been already isolated from oral cavities of healthy persons, but without taking into consideration the periodontal status [29].

The subgingival area as a reservoir of yeasts should be taken into consideration seriously, since in our study 15.7% of subjects exhibited *Candida* spp. only in subgingival biofilm, similar to the findings of Hammad et al [30]. Patients included in this study were without symptoms and medical history of candidiasis treatment. Also, in clinical praxis when candidiasis is suspected to be present, oral swabs are taken from the oral mucosa, but not from the subgingival area. Additionally, *Candida* spp. in subgingival areas is more resistant because it is always present within biofilms, and additionally these sites are inaccessible to antifungal drugs [31]. The presence of *Candida* spp. in subgingival area is important because biofilms increase resistance to conventional antifungal agents [32, 33]. In the case of inflamed periodontal tissue which means ulcerated epithelium of periodontal pocket, the entrance of microorganisms into the bloodstream is enabled. In cases of immunocompromised patients, this could potentially be life-threatening [34]. Since oral *Candida* spp. carriage may be influenced by many factors nonrelated to diabetes (gender, smoking, medications, saliva, oral site, blood type (O vs. A, AB and B blood type) [35]), we analyzed various possible factors which can be potentially confounders for *Candida* spp. carriage. To the best of our knowledge, this is the first study that took into consideration almost all factors associated with yeasts. As it is mentioned earlier, only HbA1c was predictor for the presence of Candida, but with a low risk ratio. Subgingival Candida carriage has already been connected with glycoregulation [13, 30], but results of the connection between HbA1c level and yeast detection on tongue varies [3, 19, 30]. Evidence about the role of smoking and gender on *Candida* spp. colonization and infections differ between studies (12, [20, 36, 37]. In our study, these parameters, similarly distributed between the groups, did not influence the Candida presence.

We did not find a relationship between clinical periodontal parameters and the presence of subgingival yeasts. Although it has been proven that *Candida* spp. colonization is affected by hygiene habits [38], some studies also did not find a correlation between amount of dental biofilm or gingival status with the presence of subgingival yeasts [30, 39].

Even though there is a lot of evidence about the presence of yeasts in periodontal pockets, their role in pathogenesis of periodontitis is still to be elucidated. Detection of *Candida* spp. in subgingival biofilm is only the first step for connecting yeast and periodontitis. As mentioned earlier, this presence could be transient [10], so longitudinal rather than cross sectional studies are needed for making accurate conclusions. Cross sectional design of the study, along with only one site (the deepest periodontal pocket) of subgingival biofilm collection are the main limitations of this study. Likewise, immunological response around different species and morphological forms of *Candida* spp. should be examined, but also taking into consideration all factors that could potentially affect the presence and quantity of yeasts. Variation of Candida carriage depending on the geographical region and/or patient group [40] are additional problems. In some countries, even non-albicans forms could be more frequently isolated than *C*. *albicans* [40]. In Serbia, there are not a sufficient number of studies examining the presence of different *Candida* species. A study examining prevalence of *C*. *albicans* and non-albicans species at oral lesions [41] and examining their extra oral prevalence [42] showed also highest prevalence of *C*. *albicans* [43].

As mentioned, besides the potential role in periodontitis, subgingival areas could be a potential reservoir and focus of yeasts, so the new approaches to the therapy of oral candidiasis, including obligatory scaling and root plaining should be considered.

## Conclusions

- Presence of *Candida* spp. on the tongue is higher in diabetic with poor glycoregulation and is only influenced by HbA1c serum level (glycoregulation).
- Presence of subgingival *Candida* spp. has not been influenced by parameters of oral hygiene or clinical periodontal parameters, but was influenced by HbA1c serum level.
- The presence of *Candida* spp. in subgingival areas (even when they are not found on the tongue) indicate that subgingival biofilms could be a potential reservoir of these microorganisms.

## Supporting information

S1 File Questionnare used in the study (English). (PDF)

S2 File Questionnare used in the study (Serbian). (PDF)

S3 File All collected data. (SAV)
