# Supplementary material for: Subgingival areas as potential reservoirs of different Candida spp in type 2 diabetes patients and healthy subjects
Source: PLoS One. 2019 Jan 10;14(1):e0210527. doi: 10.1371/journal.pone.0210527 (PMC6328191; doi:10.1371/journal.pone.0210527)
Supplement: S1 File — (PDF) [file pone.0210527.s001.pdf]

ANAMNESTIC CHART

Chart No:

Group:

A. **Control**
B. **Periodontitis + systematically health subjects**
C. **Periodontitis + Type 2 Diabetes (HbA1c ≤ 7.5%)**
D. **Periodontitis + Type 2 Diabetes (HbA1c > 7.5%)**

1. Demographic data

Name:	
Gender:	male□ female□
Telephone No:	
Date of birth:	
Occupation:	

2. Exclusion criteria

- ☐ antibiotics' usage in previous six month
- ☐ antifungals' usage in previous six month
- ☐ anti inflammatory therapy usage in previous six month
- ☐ scaling and root plaining in previous 1.5 year
- ☐ Period, lactation or pregnancy at the moment
- ☐ diagnose of any systemic disease except Type 2 diabetes and its chronic complication
- ☐ obesity (BMI > 30kg/m^2)
- ☐ daily usage of oral antiseptics
- ☐ presence of any disease of oral cavity except chronic periodontitis and decays
- ☐ history of malignancy
- ☐ less than 14 teeth (except third molars)
- ☐ presence of dentures

3. Personal history

☐	Menopause: □no □yes
☐	Previously treatment of oral candidiasis: □no □yes, when
☐	Blood type:
☐	Oral symptoms<br>○ Xerostomia<br>○ Sialorrhea<br>○ Stomatodynia, Stomatopyrosis<br>○ Halitosis

**3a- Type 2 diabetes groups**	**3b- Systemically health group**
Duration of diabetes in years:	Family history of diabetes
How long has the T2D be treated:	□yes □no
Therapy:	
Analysis:<br>• Fasting plasma glucose=<br>• HbA_1C=<br>• Hgb=<br>• RDC=	• MCV=<br>• MCH=<br>• MCHC=<br>• HCT=<br>• Sedimentation rate=

$3a_1$-Diabetes complications

- Retinopathy
- Nephropathy
- Neuropathy
- Macro vascular complications
  - Coronary artery disease/ infarctus myocardii
  - Coronary cerebrovascular diseases

**4. Family history**	**5. Behavioral factors**
☐ Diabetes type 1 type2	☐ Smoking*
☐ Periodontitis	☐ Daily alcohol consumption
	☐ Mouth breathing
	☐ Bruxism
	☐ Nail biting
	☐ Everyday consumption of carbohydrates

*Cigarette smoking:

- smokers
  - N<10 cigarette per day

    - $N \geq 10$ cigarette per day
- Non-smokers (never smoked cigarettes)
- Former smokers
    - Ceased smoking less than five years ago
    - Ceased smoking more than five years ago

PERIODONTAL CLINICAL EXAMINATION

**PI**														
**BOP**														
**PPD**														
**CAL**														
	**17**	**16**	**15**	**14**	**13**	**12**	**11**	**21**	**22**	**23**	**24**	**25**	**26**	**27**

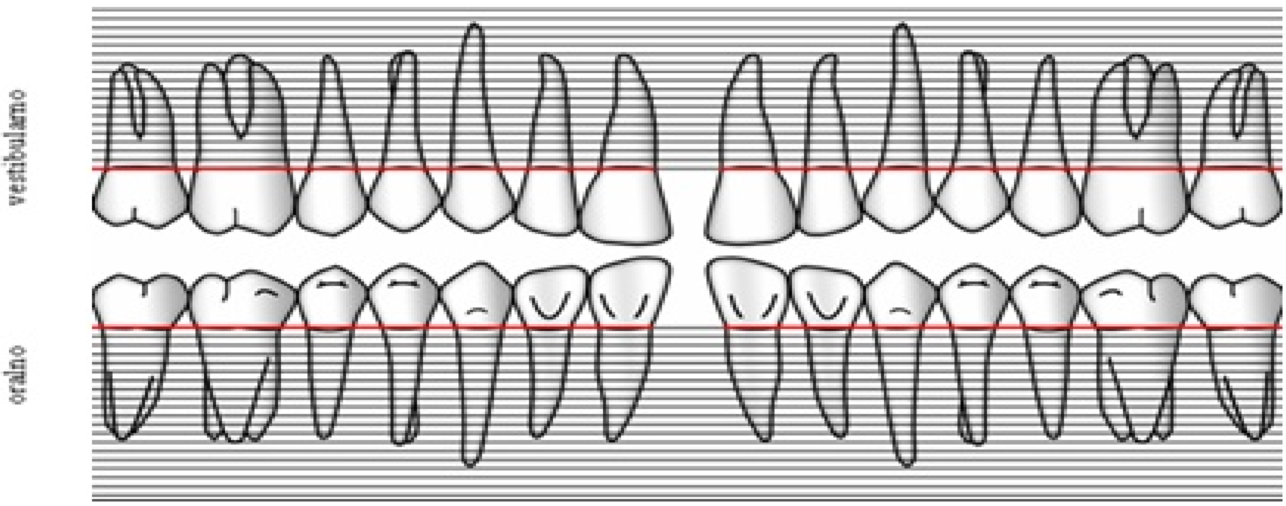

	**17**	**16**	**15**	**14**	**13**	**12**	**11**	**21**	**22**	**23**	**24**	**25**	**26**	**27**
**PI**														
**BOP**														
**PPD**														
**CAL**														

Number of present teeth (without third molars)=___________________

PI=

BOP=

PPD=

CAL=

MICROBIOLOGICAL ANALYSIS:

Cotton swabs from dorsal surface of tongue	□negative	□positive results
		CFU=
		C. albicans: □ yes □no
		C.tropicalis: □yes □no
		C.glabrata: □ yes □no
		C.parapsilosis: □ yes □no
		C.dubliniensis: □ yes □no
Subgingival plaque samples-collected by sterile paper points	□negative	□positive results
		CFU=
		C. albicans: □ yes □no
		C.tropicalis: □yes □no
		C.glabrata: □ yes □no
		C.parapsilosis: □ yes □no
		C.dubliniensis: □ yes □no
Subgingival plaque samples-collected by sterile periodontal curette	□negative	□positive results
		CFU=
		C. albicans: □ yes □no
		C.tropicalis: □yes □no
		C.glabrata: □ yes □no
		C.parapsilosis: □ yes □no
		C.dubliniensis: □ yes □no

Signature of doctor: ____________________

Signature of subjects: ______________________

Date: ____________________
