# Supplementary material for: Subgingival areas as potential reservoirs of different Candida spp in type 2 diabetes patients and healthy subjects
Source: PLoS One. 2019 Jan 10;14(1):e0210527. doi: 10.1371/journal.pone.0210527 (PMC6328191; doi:10.1371/journal.pone.0210527)
Supplement: S2 File — (PDF) [file pone.0210527.s002.pdf]

EVIDENCIONI KARTON

Karton broj: ☐

Grupa:

A. **Kontrolna**
B. **Parodontopatija+sistemski zdravi ispitanici**
C. **Parodontopatija+dm_2 (HbA1c ≤ 7.5%)**
D. **Parodontopatija+dm_2 (HbA1c > 7.5%)**

1. Generalije

Ime:	
Pol	m□ ž□
Broj telefona:	
Datum rođenja:	
Zaposlenje, zanimanje	

2. Kriterijumi za isključenje pacijenata iz studije

- ☐ antibiotici u prethodnih šest meseci
- ☐ antimikotici u prethodnih šest meseci
- ☐ antiinflamatorni lekovi u prethodna šest meseci
- ☐ terapija parodontopatije u prethodnih godinu i po dana
- ☐ trenutno menstruacija, dojenje, trudnoća
- ☐ prisustvo sistemskih oboljenja osim dijabetesa i hroničnih komlikacija dijabetesa
- ☐ gojaznost (BMI index > $30kg/m^2$)
- ☐ svakodnevna upotreba lokalnih oralnih antiseptika
- ☐ prisustvo bilo kog oralnog oboljenja sem karijesa i parodontopatije
- ☐ istorija bilo kog maligniteta
- ☐ manje od 14 zuba (ne računaju se treći molari)
- ☐ prisustvo mobilnih nadoknada u ustima

3. Lična anamneza

☐	Menopauza: □ne □da, od kada
☐	Istorija terapije oralne kandidioze: □ne □da, navedi
☐	Krvna grupa:
☐	Simptomi u usnoj duplji<br>o Kserostomija<br>o Sijaloreja<br>o Peckanje, žarenje, bol<br>o Zadah

3a- Dijabetes melitus grupa **$3a_1$- sistemski zdravi ispitanici**

Godina dijagnostikovanja dijabetesa:	Dijabetes u porodičnoj anamnezi
Godina početka terapije:	□da □ne
Terapija:	
Analize<br>• Glukoza natašte<br>• HbA_1C=<br>• Hb=<br>• Er=	• MCV=<br>• MCH=<br>• MCHC=<br>• HCT=<br>• Sedimentacija=

$3a_2$-Komplikacije dijabetesa

- Retinopatija
- Nefropatija
- Periferna neuropatija
- Makrovaskularne komplikacije
  - Oboljenja koronarnih ks/infarkt miokarda
  - Cerebrovaskularna oboljenja/šlog

4. Porodična anamneza

5. Loše navike

4. Porodična anamneza	5. Loše navike
☐ Dijabetes tip 1 tip2	☐ Pušenje*
☐ Parodontopatija A C	☐ Alkohol
	☐ Disanje na usta
	☐ Bruksizam/stiskanje zuba
	☐ Grickanje stranih predmeta
	☐ Svakodnevna konzumacija slatkiša

*Pušenje cigareta:

- pušači
  - N<10 cigareta na dan

  - N≥10 cigareta na dan
- nepušači (nikad nisu pušili cigare)
- bivči pušači
  - ostavili pušenje pre manje od 5 godina
  - ostavili pušenje pre više od 5 godina

KLINIČKA MERENJA I REZULTATI KLINIČKOG PREGLEDA

**PI**														
**KNP**														
**DS**														
**NPE**														
	**17**	**16**	**15**	**14**	**13**	**12**	**11**	**21**	**22**	**23**	**24**	**25**	**26**	**27**

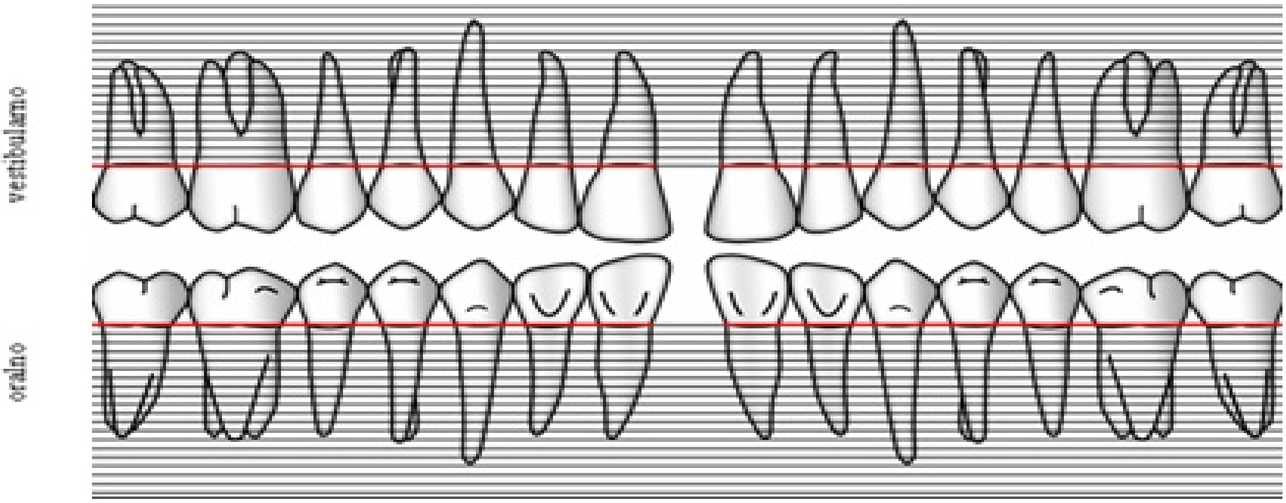

	**17**	**16**	**15**	**14**	**13**	**12**	**11**	**21**	**22**	**23**	**24**	**25**	**26**	**27**
**PI**														
**KNP**														
**DS**														
**NPE**														

Broj zuba u ustima (bez trećih molara)=___________________

PI=

KNP=

DS=

NPE=

REZULTATI MIKROBIOLOŠKIH ANALIZA:

Bris jezika	□negativan	□pozitivan
		CFU=
		C. albicans: □ da □ne
		C.tropicalis: □ da □ne
		C.glabrata: □ da □ne
		C.parapsilosis: □ da □ne
		C.dubliniensis: □ da □ne
Uzorak subgingivalnog plaka-sterilni papirni poen	□negativan	□pozitivan
		CFU=
		C. albicans: □ da □ne
		C.tropicalis: □ da □ne
		C.glabrata: □ da □ne
		C.parapsilosis: □ da □ne
		C.dubliniensis: □ da □ne
Uzorak subgingivalnog plaka-sterilna parodontalna kireta	□negativan	□pozitivan
		CFU=
		C. albicans: □ da □ne
		C.tropicalis: □ da □ne
		C.glabrata: □ da □ne
		C.parapsilosis: □ da □ne
		C.dubliniensis: □ da □ne

Uzorke uzeo: Potpis ispitanika:

___________________ ______________________

Datum:

____
